# Bis[1-(isopropyl­ideneamino)guanidinium] bis­(3-nitro­benzoate) monohydrate

**DOI:** 10.1107/S1600536809048612

**Published:** 2009-11-25

**Authors:** Janet M. S. Skakle, Edward R. T. Tiekink, James L. Wardell, Solange M. S. V. Wardell

**Affiliations:** a Department of Chemistry, University of Aberdeen, Old Aberdeen AB15 5NY, Scotland; b Department of Chemistry, University of Malaya, 50603 Kuala Lumpur, Malaysia; c Centro de Desenvolvimento Tecnológico em Saúde (CDTS), Fundação Oswaldo Cruz (FIOCRUZ), Casa Amarela, Campus de Manguinhos, Av. Brasil 4365, 21040-900 Rio de Janeiro, RJ, Brazil; d CHEMSOL, 1 Harcourt Road, Aberdeen AB15 5NY, Scotland

## Abstract

The asymmetric unit of the title salt hydrate, 2C_4_H_11_N_4_^+^·2C_7_H_4_NO_4_^−^·H_2_O, comprises two independent 1-(isopropyl­ideneamino)guanidinium cations, two independent 3-nitro­benzoate anions and a water mol­ecule of crystallization. There are minimal geometric differences between the two planar [maximum deviations 0.061 (2) and 0.088 (2) Å] cations, and between the two almost planar anions [C–C–C–O and C–C–N–O torsion angles of 0.3 (3) and 11.1 (4) °, respectively in the first anion and −173.7 (2) and −0.1 (4), respectively in the second anion]. Extensive O—H⋯O and N—H⋯O hydrogen bonding between all components of the structure leads to the formation of a two-dimensional array with an undulating topology in the *bc* plane.

## Related literature

For the structure of 1-(isopropyl­ideneamino)guanidinium 2-nitro­benzoate, see: Skakle *et al.* (2006 ▶).
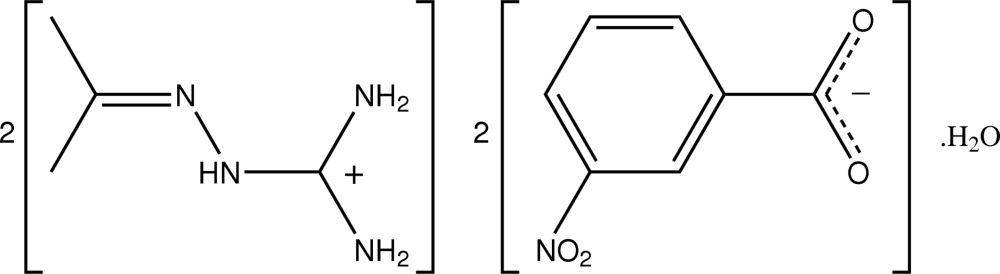


## Experimental

### 

#### Crystal data

2C_4_H_11_N_4_^+^·2C_7_H_4_NO_4_^−^·H_2_O
*M* *_r_* = 580.58
Monoclinic, 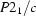

*a* = 16.5833 (6) Å
*b* = 22.2457 (10) Å
*c* = 7.5424 (3) Å
β = 92.232 (2)°
*V* = 2780.33 (19) Å^3^
*Z* = 4
Mo *K*α radiation
μ = 0.11 mm^−1^
*T* = 120 K
0.20 × 0.08 × 0.06 mm

#### Data collection

Enraf–Nonius KappaCCD area-detector diffractometer
Absorption correction: multi-scan (*SADABS*; Sheldrick, 2003 ▶) *T*_min_ = 0.041, *T*_max_ = 0.099
29694 measured reflections
6339 independent reflections
3607 reflections with *I* > 2σ(*I*)
*R*_int_ = 0.072

#### Refinement

*R*[*F* ^2^ > 2σ(*F* ^2^)] = 0.053
*wR*(*F* ^2^) = 0.177
*S* = 1.06
6339 reflections
380 parameters
3 restraints
H-atom parameters constrained
Δρ_max_ = 0.35 e Å^−3^
Δρ_min_ = −0.56 e Å^−3^

### 

Data collection: *COLLECT* (Hooft, 1998 ▶); cell refinement: *DENZO* (Otwinowski & Minor, 1997 ▶) and *COLLECT*; data reduction: *DENZO* and *COLLECT*; program(s) used to solve structure: *SHELXS97* (Sheldrick, 2008 ▶); program(s) used to refine structure: *SHELXL97* (Sheldrick, 2008 ▶); molecular graphics: *DIAMOND* (Brandenburg, 2006 ▶); software used to prepare material for publication: *publCIF* (Westrip, 2009 ▶).

## Supplementary Material

Crystal structure: contains datablocks global, I. DOI: 10.1107/S1600536809048612/hg2595sup1.cif

Structure factors: contains datablocks I. DOI: 10.1107/S1600536809048612/hg2595Isup2.hkl

Additional supplementary materials:  crystallographic information; 3D view; checkCIF report

## Figures and Tables

**Table 1:** Hydrogen-bond geometry (Å, °)

*D*—H⋯*A*	*D*—H	H⋯*A*	*D*⋯*A*	*D*—H⋯*A*
O1w—H1w⋯O1	0.84	2.06	2.878 (2)	164
O1w—H2w⋯O5	0.84	1.93	2.755 (2)	168
N3—H3*A*⋯O2	0.88	1.88	2.755 (3)	170
N3—H3*B*⋯O6^i^	0.88	2.01	2.812 (3)	150
N4—H4*A*⋯O1	0.88	2.06	2.943 (3)	177
N4—H4*B*⋯O5	0.88	2.32	3.049 (3)	139
N5—H5*A*⋯O6^i^	0.88	2.13	2.880 (3)	142
N7—H7*A*⋯O2^i^	0.88	2.04	2.779 (3)	141
N7—H7*B*⋯O1	0.88	2.07	2.831 (3)	144
N8—H8*A*⋯O1w^ii^	0.88	2.03	2.881 (3)	161
N8—H8*B*⋯O1w^iii^	0.88	2.13	2.975 (3)	162
N9—H9⋯O5^iii^	0.88	2.19	2.904 (3)	138
C5—H5⋯O7^iv^	0.95	2.58	3.322 (3)	135
C13—H13⋯O3^iii^	0.95	2.57	3.233 (4)	127
C21—H21*B*⋯O8^v^	0.98	2.34	3.290 (3)	164
C22—H22*B*⋯O6^vi^	0.98	2.57	3.538 (3)	168

Symmetry codes: (i) 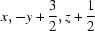; (ii) 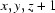; (iii) 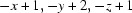; (iv) 
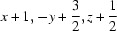
; (v) 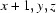; (vi) 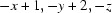.

**Fig. 1.**
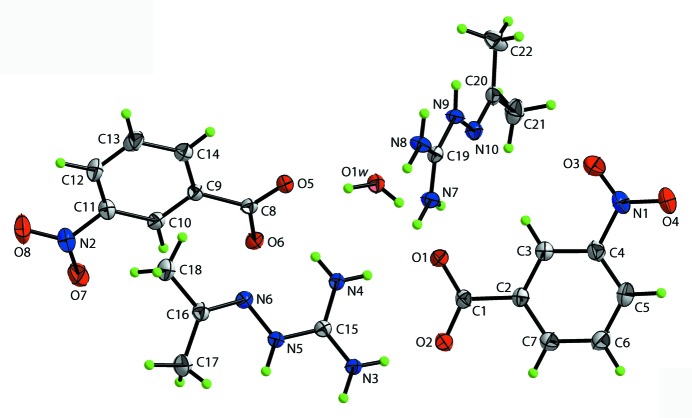
Molecular structures of the components of the asymmetric unit in (I) showing atom-labelling scheme and displacement ellipsoids at the 50% probability level.

**Fig. 2.**
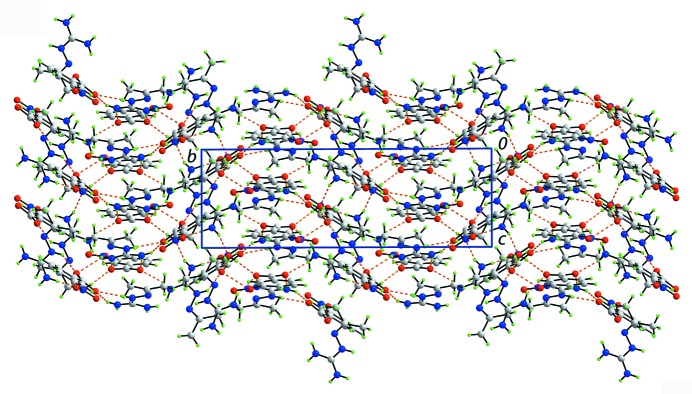
A plan view of the 2-D array in the bc plane in the crystal structure of (I). The O—H···O and N—H···O hydrogen bonding is shown as orange dashed lines.

**Fig. 3.**
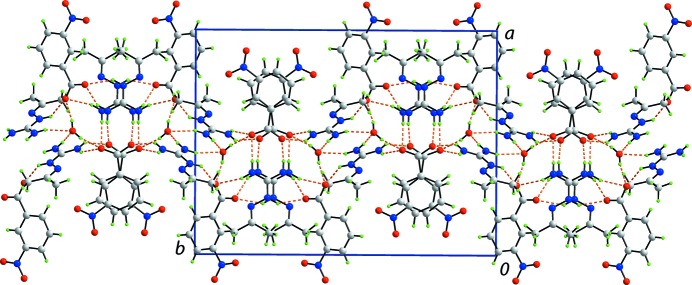
A side-on view of the 2-D array in the bc plane in the crystal structure of (I) highlighting the undulating topology. The O—H···O and N—H···O hydrogen bonding is shown as orange dashed lines.

**Fig. 4.**
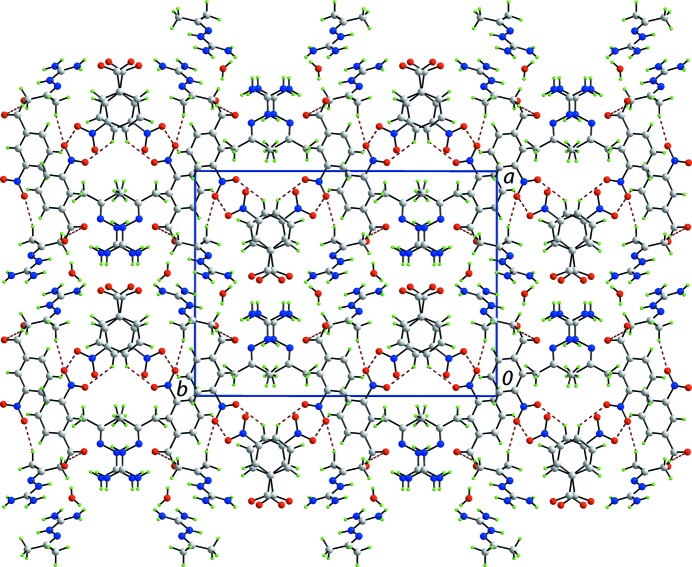
A view in projection down the c axis showing the stacking of layers in the crystal structure of (I). Layers are connected by C—H···O contacts, shown as brown dashed lines.

## supplementary crystallographic information

### Comment

Following on the recent publication of the structure of 1-(isopropyplideneamino)guanidinium 2-nitrobenzoate (Skakle *et al.*, 2006), we now report the structure of the related salt, 1-(isopropyplideneamino)guanidinium 3-nitrobenzoate, obtained as a hydrate, (I). The crystallographic asymmetric unit of (I) comprises two independent 1-(isopropylideneamino)guanidinium cations, two independent 3-nitrobenzoate anions, and a water molecule of crystallization. Confirmation of proton transfer from the benzoic acid derivative to the guanine molecule during crystallization is found in the equivalence of the C—O bond distances (C1—O1, O2 = 1.259 (3) and 1.258 (3) Å; C8—O5, O6 = 1.261 (3) and 1.255 (3) Å) and the pattern of hydrogen bonding, see below. The guanidinium cations are virtually identical as seen in the r.m.s. values for bond distances and angles of 0.012 Å and 1.38 °, respectively. In the same way, the two independent anions have very similar geometries as indicated by the r.m.s. values for bond distances and angles of 0.005 Å and 1.06 °, respectively. The maximum deviation from planarity of the eight non-hydrogen atoms in the C15-containing cation is 0.061 (2) Å for atom N3. For the C19 cation, the maximum deviation of 0.088 (2) Å is also found for an amino group, *i.e.* atom N7. The benzene rings of the anions are also planar with the carboxylate and nitro groups co-planar and slightly twisted, respectively, out of the plane of the C2—C7 benzene ring as seen in the C3–C2–C1–O1 and C3–C4–N1–O3 torsion angles of 0.3 (3) ° and 11.1 (4) °, respectively. The second independent anion is even more planar: the C10–C9–C8–O5 and C10–C11—N2–O7 torsion angles are -173.7 (2) and -0.1 (4), respectively.

Not surprisingly from the constitution of (I), there is extensive hydrogen bonding at play in the crystal structure. The most prominent intermolecular interactions are of the type O—H···O and N—H···O, Table 1. The water molecule provides two donor interactions to two benzoate-O atoms and accepts two hydrogen bond from two N8-amino groups derived from two different cations. The later interactions occur around a centre of inversion and results in the formation of eight-membered {···HNH···O}_2_ synthons. The C15-guanidinium cation utilizes all five acidic-H atoms to hydrogen bond to four oxygen atoms, derived from three symmetry related benzoate anions. One H atom from each of the N3- and N4-amino groups each connects an O atom of an O1-benzoate thereby forming an eight-membered {···HNCNH···OCO} synthon. The other N3—H atom hydrogen bonds to a benzoate-O6 atom which at the same time accepts a hydrogen bond from the N5—H atom to close a S(6) ring. The other N4—H atom hydrogen bonds to a benzoate-O5 atom so that the amino-N4—H_2_ bridges the same two benzoate-O atoms as does the water molecule to form an eight-membered {···HNH···O···HOH···O} synthon.

The C19-guanidinium cation also connects to five O atoms, three derived from three symmetry related benzoate groups and two water molecules; the N8—H_2_ amino group bridges two water molecules, as described above. Each of amino-N7 H atoms is connected to a symmetry related O1-benzoate anion so that rows of benzoate anions along the *c* axis are bridged by amino-N7—H_2_ groups. Finally, the N9—H links a benzoate-O5 atom. Each of the imine-N6 and –N10 atoms participates in intramolecular interactions with the N4- and N7—H atoms, respectively. The O1-benzoate forms five acceptor interactions, one from the water molecule and four from N—H. The O5-benzoate forms the same number and type of acceptor interactions. The net result of the O—H···O and N—H···O hydrogen bonding is the formation of a 2-D array in the *bc* plane, Fig. 2. These have an undulating topology, Fig. 3. The layers stack along the *a* direction being held in place by C—H···O contacts where the O atoms are derived from benzoate (O6) and nitro (O3, O7, O8) O atoms, Table 1 and Fig. 4.

An undulating 2-D array was found in the crystal structure of anhydrous 1-(isopropylideneamino)guanidinium 2-nitrobenzoate, see: Skakle *et al.* (2006).

### Experimental

A solution of aminoguanidinium carbonate (0.290 g, 2.1 mmol) in MeOH (15 ml) was added to a solution of 3-nitrobenzoic acid (0.350 g, 2.1 mmol) in MeOH (15 ml) and mixed. After the effervescence had subsided, the reaction mixture was refluxed for 15 min, left at room temperature overnight, and then rotary evaporated to leave a residue of [(H_2_N)_2_CNHNH_2_][3-O_2_NC_6_H_4_CO_2_]. The crude material was dissolved in acetone and the solution left at room temperature to produce crystals characterized as (I), m. pt. 436–435 K.

### Refinement

The N– and C-bound H atoms were geometrically placed with N—H = 0.88 Å and C—H = 0.95–0.98 Å, and refined as riding with *U_iso_*(H) = 1.2–1.*5U_eq_*(C). A rotating group model was used for the methyl groups. The water-bound H atoms were located from a difference map and included in the model with restraints O–H = 0.840±0. 001 Å and H1w···Hw2 = 1.39±0.01, and with *U_iso_*(H) = 1.*5U_eq_*(O).

### Crystal data

**Table:**

2C_4_H_11_N_4_^+^·2C_7_H_4_NO_4_^−^·H_2_O	*F*(000) = 1224
*M**_r_* = 580.58	*D*_x_ = 1.387 Mg m^−^^3^
Monoclinic, *P*2_1_/*c*	Mo *K*α radiation, λ = 0.71073 Å
Hall symbol: -P 2ybc	Cell parameters from 6191 reflections
*a* = 16.5833 (6) Å	θ = 1.0–27.5°
*b* = 22.2457 (10) Å	µ = 0.11 mm^−^^1^
*c* = 7.5424 (3) Å	*T* = 120 K
β = 92.232 (2)°	Prism, colourless
*V* = 2780.33 (19) Å^3^	0.20 × 0.08 × 0.06 mm
*Z* = 4	

### Data collection

**Table:**

Enraf–Nonius KappaCCD area-detector diffractometer	6339 independent reflections
Radiation source: Enraf Nonius FR591 rotating anode	3607 reflections with *I* > 2σ(*I*)
10 cm confocal mirrors	*R*_int_ = 0.072
Detector resolution: 9.091 pixels mm^-1^	θ_max_ = 27.5°, θ_min_ = 1.2°
φ and ω scans	*h* = −21→21
Absorption correction: multi-scan (*SADABS*; Sheldrick, 2003)	*k* = −26→28
*T*_min_ = 0.041, *T*_max_ = 0.099	*l* = −9→9
29694 measured reflections	

### Refinement

**Table:**

Refinement on *F*^2^	Primary atom site location: structure-invariant direct methods
Least-squares matrix: full	Secondary atom site location: difference Fourier map
*R*[*F*^2^ > 2σ(*F*^2^)] = 0.053	Hydrogen site location: inferred from neighbouring sites
*wR*(*F*^2^) = 0.177	H-atom parameters constrained
*S* = 1.06	*w* = 1/[σ^2^(*F*_o_^2^) + (0.0941*P*)^2^] where *P* = (*F*_o_^2^ + 2*F*_c_^2^)/3
6339 reflections	(Δ/σ)_max_ < 0.001
380 parameters	Δρ_max_ = 0.35 e Å^−^^3^
3 restraints	Δρ_min_ = −0.56 e Å^−^^3^

### Special details

**Table:**

Geometry. All s.u.'s (except the s.u. in the dihedral angle between two l.s. planes) are estimated using the full covariance matrix. The cell s.u.'s are taken into account individually in the estimation of s.u.'s in distances, angles and torsion angles; correlations between s.u.'s in cell parameters are only used when they are defined by crystal symmetry. An approximate (isotropic) treatment of cell s.u.'s is used for estimating s.u.'s involving l.s. planes.
Refinement. Refinement of *F*^2^ against ALL reflections. The weighted *R*-factor *wR* and goodness of fit *S* are based on *F*^2^, conventional *R*-factors *R* are based on *F*, with *F* set to zero for negative *F*^2^. The threshold expression of *F*^2^ > 2σ(*F*^2^) is used only for calculating *R*-factors(gt) *etc*. and is not relevant to the choice of reflections for refinement. *R*-factors based on *F*^2^ are statistically about twice as large as those based on *F*, and *R*- factors based on ALL data will be even larger.

### Fractional atomic coordinates and isotropic or equivalent isotropic displacement parameters (Å^2^)

**Table:**

	*x*	*y*	*z*	*U*_iso_*/*U*_eq_	
O1	0.53214 (10)	0.81252 (8)	0.2856 (2)	0.0255 (4)	
O2	0.51509 (10)	0.71297 (8)	0.2780 (2)	0.0320 (5)	
O3	0.80185 (12)	0.89030 (10)	0.4371 (3)	0.0526 (6)	
O4	0.90614 (12)	0.83281 (11)	0.4219 (4)	0.0656 (7)	
N1	0.83303 (14)	0.84104 (12)	0.4178 (3)	0.0426 (6)	
C1	0.55819 (14)	0.75937 (12)	0.2933 (3)	0.0219 (6)	
C2	0.64799 (14)	0.74986 (11)	0.3288 (3)	0.0222 (6)	
C3	0.69888 (15)	0.79915 (12)	0.3527 (3)	0.0247 (6)	
H3	0.6783	0.8390	0.3448	0.030*	
C4	0.77989 (15)	0.78874 (13)	0.3881 (3)	0.0285 (6)	
C5	0.81320 (16)	0.73169 (13)	0.3984 (4)	0.0327 (7)	
H5	0.8695	0.7262	0.4200	0.039*	
C6	0.76174 (16)	0.68290 (13)	0.3763 (4)	0.0338 (7)	
H6	0.7825	0.6432	0.3850	0.041*	
C7	0.68044 (15)	0.69216 (12)	0.3418 (3)	0.0288 (6)	
H7	0.6457	0.6584	0.3265	0.035*	
O8	−0.11247 (11)	0.93385 (11)	0.1007 (3)	0.0561 (6)	
O7	−0.03790 (12)	0.86476 (10)	−0.0110 (3)	0.0499 (6)	
O6	0.24821 (10)	0.86769 (8)	0.0634 (2)	0.0280 (4)	
O5	0.31194 (9)	0.93239 (8)	0.2451 (2)	0.0240 (4)	
N2	−0.04684 (13)	0.91043 (12)	0.0778 (3)	0.0384 (6)	
C8	0.24919 (14)	0.91177 (11)	0.1672 (3)	0.0214 (5)	
C9	0.16892 (14)	0.94107 (11)	0.1993 (3)	0.0219 (5)	
C10	0.09944 (14)	0.91448 (12)	0.1272 (3)	0.0253 (6)	
H10	0.1030	0.8790	0.0578	0.030*	
C11	0.02547 (14)	0.93963 (12)	0.1564 (3)	0.0286 (6)	
C12	0.01764 (16)	0.99132 (13)	0.2560 (4)	0.0338 (7)	
H12	−0.0341	1.0078	0.2759	0.041*	
C13	0.08689 (16)	1.01847 (13)	0.3262 (4)	0.0340 (7)	
H13	0.0829	1.0542	0.3943	0.041*	
C14	0.16210 (15)	0.99375 (12)	0.2974 (3)	0.0275 (6)	
H14	0.2093	1.0129	0.3451	0.033*	
N3	0.36556 (12)	0.70834 (10)	0.4243 (3)	0.0270 (5)	
H3A	0.4153	0.7070	0.3880	0.032*	
H3B	0.3406	0.6749	0.4518	0.032*	
N4	0.36412 (12)	0.81090 (10)	0.3975 (3)	0.0255 (5)	
H4A	0.4139	0.8106	0.3609	0.031*	
H4B	0.3381	0.8452	0.4073	0.031*	
N5	0.25179 (11)	0.75952 (9)	0.4934 (3)	0.0241 (5)	
H5A	0.2268	0.7253	0.5121	0.029*	
N6	0.21349 (12)	0.81397 (9)	0.5205 (3)	0.0238 (5)	
C15	0.32863 (14)	0.76011 (11)	0.4374 (3)	0.0216 (5)	
C16	0.14172 (14)	0.81102 (12)	0.5791 (3)	0.0237 (6)	
C17	0.09737 (16)	0.75369 (13)	0.6148 (4)	0.0331 (7)	
H17A	0.0884	0.7315	0.5035	0.050*	
H17B	0.0453	0.7631	0.6651	0.050*	
H17C	0.1295	0.7291	0.6990	0.050*	
C18	0.09902 (16)	0.86897 (13)	0.6092 (4)	0.0335 (7)	
H18A	0.1345	0.9026	0.5810	0.050*	
H18B	0.0845	0.8716	0.7337	0.050*	
H18C	0.0500	0.8708	0.5326	0.050*	
N7	0.53461 (12)	0.89118 (9)	0.5808 (3)	0.0249 (5)	
H7A	0.5064	0.8672	0.6476	0.030*	
H7B	0.5489	0.8791	0.4755	0.030*	
N8	0.53482 (12)	0.96461 (9)	0.7968 (3)	0.0250 (5)	
H8A	0.5066	0.9415	0.8660	0.030*	
H8B	0.5495	1.0008	0.8325	0.030*	
N9	0.59766 (12)	0.98184 (9)	0.5355 (3)	0.0228 (5)	
H9	0.6083	1.0193	0.5655	0.027*	
N10	0.62350 (12)	0.95652 (9)	0.3776 (3)	0.0235 (5)	
C19	0.55533 (14)	0.94484 (11)	0.6384 (3)	0.0210 (5)	
C20	0.66388 (14)	0.99055 (12)	0.2777 (3)	0.0242 (6)	
C21	0.69303 (16)	0.96204 (14)	0.1118 (3)	0.0356 (7)	
H21A	0.6731	0.9206	0.1033	0.053*	
H21B	0.7522	0.9619	0.1154	0.053*	
H21C	0.6729	0.9850	0.0083	0.053*	
C22	0.68386 (17)	1.05488 (13)	0.3123 (4)	0.0355 (7)	
H22A	0.6343	1.0771	0.3352	0.053*	
H22B	0.7092	1.0720	0.2085	0.053*	
H22C	0.7212	1.0578	0.4159	0.053*	
O1W	0.45485 (10)	0.90793 (8)	0.0851 (2)	0.0270 (4)	
H1W	0.4854	0.8820	0.1333	0.041*	
H2W	0.4102	0.9100	0.1336	0.041*	

### Atomic displacement parameters (Å^2^)

**Table:**

	*U*^11^	*U*^22^	*U*^33^	*U*^12^	*U*^13^	*U*^23^
O1	0.0233 (9)	0.0224 (11)	0.0309 (9)	0.0022 (7)	0.0003 (7)	−0.0020 (8)
O2	0.0231 (9)	0.0249 (11)	0.0485 (11)	−0.0021 (8)	0.0076 (8)	−0.0120 (9)
O3	0.0361 (12)	0.0316 (13)	0.0890 (17)	−0.0050 (10)	−0.0122 (12)	−0.0060 (12)
O4	0.0224 (12)	0.0513 (16)	0.122 (2)	−0.0042 (10)	−0.0123 (12)	0.0050 (15)
N1	0.0237 (13)	0.0381 (17)	0.0651 (17)	−0.0057 (12)	−0.0076 (12)	0.0023 (13)
C1	0.0208 (13)	0.0239 (15)	0.0213 (12)	−0.0004 (11)	0.0050 (10)	−0.0049 (10)
C2	0.0221 (13)	0.0243 (15)	0.0205 (12)	0.0010 (10)	0.0016 (10)	−0.0010 (10)
C3	0.0229 (13)	0.0246 (15)	0.0266 (13)	0.0004 (11)	0.0010 (11)	0.0002 (11)
C4	0.0220 (13)	0.0323 (17)	0.0310 (14)	−0.0018 (12)	−0.0025 (11)	0.0013 (12)
C5	0.0249 (14)	0.0386 (18)	0.0343 (15)	0.0059 (13)	−0.0035 (12)	0.0016 (13)
C6	0.0333 (16)	0.0278 (17)	0.0399 (15)	0.0071 (13)	−0.0040 (13)	0.0006 (12)
C7	0.0279 (14)	0.0265 (16)	0.0317 (14)	0.0000 (11)	−0.0014 (12)	−0.0015 (11)
O8	0.0191 (11)	0.0653 (17)	0.0839 (17)	0.0052 (11)	0.0019 (11)	−0.0086 (13)
O7	0.0302 (11)	0.0425 (15)	0.0761 (15)	−0.0055 (10)	−0.0088 (11)	−0.0168 (12)
O6	0.0258 (10)	0.0232 (11)	0.0354 (10)	0.0020 (8)	0.0049 (8)	−0.0071 (8)
O5	0.0189 (9)	0.0230 (10)	0.0302 (9)	−0.0009 (7)	0.0003 (7)	−0.0001 (7)
N2	0.0213 (13)	0.0424 (17)	0.0516 (15)	−0.0009 (11)	0.0009 (11)	0.0014 (13)
C8	0.0216 (13)	0.0179 (14)	0.0248 (12)	0.0013 (10)	0.0028 (11)	0.0036 (11)
C9	0.0213 (12)	0.0195 (14)	0.0252 (12)	0.0018 (10)	0.0037 (10)	0.0012 (10)
C10	0.0255 (14)	0.0202 (14)	0.0304 (13)	0.0027 (11)	0.0027 (11)	0.0003 (11)
C11	0.0194 (13)	0.0305 (16)	0.0358 (14)	0.0001 (11)	0.0016 (11)	0.0014 (12)
C12	0.0231 (14)	0.0344 (17)	0.0442 (16)	0.0079 (12)	0.0059 (13)	0.0002 (13)
C13	0.0319 (15)	0.0288 (16)	0.0414 (15)	0.0075 (12)	0.0039 (13)	−0.0101 (13)
C14	0.0241 (13)	0.0252 (15)	0.0333 (13)	0.0008 (11)	0.0007 (11)	−0.0025 (12)
N3	0.0219 (11)	0.0174 (12)	0.0425 (13)	0.0011 (9)	0.0099 (10)	0.0044 (10)
N4	0.0187 (10)	0.0208 (12)	0.0375 (12)	0.0029 (9)	0.0065 (9)	0.0043 (9)
N5	0.0203 (11)	0.0177 (12)	0.0348 (12)	−0.0009 (9)	0.0054 (9)	0.0004 (9)
N6	0.0217 (11)	0.0199 (12)	0.0300 (11)	0.0009 (9)	0.0023 (9)	−0.0009 (9)
C15	0.0205 (13)	0.0196 (14)	0.0245 (12)	0.0008 (11)	0.0014 (10)	0.0017 (10)
C16	0.0208 (13)	0.0254 (15)	0.0250 (13)	0.0018 (11)	0.0019 (10)	−0.0017 (11)
C17	0.0279 (15)	0.0336 (17)	0.0384 (15)	0.0005 (12)	0.0086 (12)	−0.0007 (13)
C18	0.0270 (14)	0.0324 (17)	0.0415 (16)	0.0031 (12)	0.0075 (13)	−0.0016 (13)
N7	0.0275 (12)	0.0198 (12)	0.0278 (11)	−0.0032 (9)	0.0075 (9)	−0.0017 (9)
N8	0.0289 (12)	0.0219 (12)	0.0247 (11)	−0.0077 (9)	0.0056 (9)	−0.0014 (9)
N9	0.0258 (11)	0.0160 (11)	0.0271 (11)	−0.0042 (9)	0.0064 (9)	−0.0005 (9)
N10	0.0228 (11)	0.0242 (12)	0.0239 (11)	0.0010 (9)	0.0043 (9)	−0.0004 (9)
C19	0.0178 (12)	0.0203 (14)	0.0247 (12)	0.0013 (10)	−0.0012 (10)	0.0027 (11)
C20	0.0153 (12)	0.0289 (16)	0.0283 (13)	0.0019 (11)	0.0003 (10)	0.0063 (11)
C21	0.0306 (15)	0.0459 (19)	0.0309 (14)	0.0076 (13)	0.0069 (12)	0.0053 (13)
C22	0.0384 (16)	0.0370 (18)	0.0311 (14)	−0.0112 (13)	0.0030 (13)	0.0060 (12)
O1W	0.0215 (9)	0.0268 (11)	0.0330 (10)	0.0019 (8)	0.0047 (8)	0.0037 (8)

### Geometric parameters (Å, °)

**Table:**

O1—C1	1.259 (3)	N4—C15	1.314 (3)
O2—C1	1.258 (3)	N4—H4A	0.8800
O3—N1	1.223 (3)	N4—H4B	0.8800
O4—N1	1.225 (3)	N5—C15	1.358 (3)
N1—C4	1.471 (4)	N5—N6	1.387 (3)
C1—C2	1.517 (3)	N5—H5A	0.8800
C2—C3	1.391 (3)	N6—C16	1.287 (3)
C2—C7	1.394 (4)	C16—C18	1.492 (4)
C3—C4	1.379 (4)	C16—C17	1.502 (4)
C3—H3	0.9500	C17—H17A	0.9800
C4—C5	1.385 (4)	C17—H17B	0.9800
C5—C6	1.387 (4)	C17—H17C	0.9800
C5—H5	0.9500	C18—H18A	0.9800
C6—C7	1.379 (4)	C18—H18B	0.9800
C6—H6	0.9500	C18—H18C	0.9800
C7—H7	0.9500	N7—C19	1.311 (3)
O8—N2	1.225 (3)	N7—H7A	0.8800
O7—N2	1.229 (3)	N7—H7B	0.8800
O6—C8	1.255 (3)	N8—C19	1.330 (3)
O5—C8	1.261 (3)	N8—H8A	0.8800
N2—C11	1.468 (3)	N8—H8B	0.8800
C8—C9	1.510 (3)	N9—C19	1.347 (3)
C9—C10	1.387 (3)	N9—N10	1.400 (3)
C9—C14	1.393 (4)	N9—H9	0.8800
C10—C11	1.374 (3)	N10—C20	1.276 (3)
C10—H10	0.9500	C20—C22	1.490 (4)
C11—C12	1.383 (4)	C20—C21	1.499 (4)
C12—C13	1.384 (4)	C21—H21A	0.9800
C12—H12	0.9500	C21—H21B	0.9800
C13—C14	1.388 (4)	C21—H21C	0.9800
C13—H13	0.9500	C22—H22A	0.9800
C14—H14	0.9500	C22—H22B	0.9800
N3—C15	1.310 (3)	C22—H22C	0.9800
N3—H3A	0.8800	O1W—H1W	0.8400
N3—H3B	0.8800	O1W—H2W	0.8399
			
O3—N1—O4	123.6 (3)	H4A—N4—H4B	120.0
O3—N1—C4	118.2 (2)	C15—N5—N6	118.6 (2)
O4—N1—C4	118.2 (3)	C15—N5—H5A	120.7
O2—C1—O1	125.0 (2)	N6—N5—H5A	120.7
O2—C1—C2	116.9 (2)	C16—N6—N5	116.2 (2)
O1—C1—C2	118.1 (2)	N3—C15—N4	121.6 (2)
C3—C2—C7	119.1 (2)	N3—C15—N5	117.5 (2)
C3—C2—C1	119.9 (2)	N4—C15—N5	120.9 (2)
C7—C2—C1	121.0 (2)	N6—C16—C18	117.3 (2)
C4—C3—C2	118.3 (2)	N6—C16—C17	124.8 (2)
C4—C3—H3	120.8	C18—C16—C17	117.9 (2)
C2—C3—H3	120.8	C16—C17—H17A	109.5
C3—C4—C5	123.2 (3)	C16—C17—H17B	109.5
C3—C4—N1	118.0 (2)	H17A—C17—H17B	109.5
C5—C4—N1	118.7 (2)	C16—C17—H17C	109.5
C4—C5—C6	117.9 (2)	H17A—C17—H17C	109.5
C4—C5—H5	121.0	H17B—C17—H17C	109.5
C6—C5—H5	121.0	C16—C18—H18A	109.5
C7—C6—C5	119.9 (3)	C16—C18—H18B	109.5
C7—C6—H6	120.1	H18A—C18—H18B	109.5
C5—C6—H6	120.1	C16—C18—H18C	109.5
C6—C7—C2	121.5 (3)	H18A—C18—H18C	109.5
C6—C7—H7	119.2	H18B—C18—H18C	109.5
C2—C7—H7	119.2	C19—N7—H7A	120.0
O8—N2—O7	123.7 (2)	C19—N7—H7B	120.0
O8—N2—C11	118.1 (3)	H7A—N7—H7B	120.0
O7—N2—C11	118.1 (2)	C19—N8—H8A	120.0
O6—C8—O5	124.4 (2)	C19—N8—H8B	120.0
O6—C8—C9	116.6 (2)	H8A—N8—H8B	120.0
O5—C8—C9	119.0 (2)	C19—N9—N10	115.4 (2)
C10—C9—C14	118.9 (2)	C19—N9—H9	122.3
C10—C9—C8	118.5 (2)	N10—N9—H9	122.3
C14—C9—C8	122.6 (2)	C20—N10—N9	116.7 (2)
C11—C10—C9	119.8 (2)	N7—C19—N8	121.7 (2)
C11—C10—H10	120.1	N7—C19—N9	120.0 (2)
C9—C10—H10	120.1	N8—C19—N9	118.2 (2)
C10—C11—C12	122.0 (2)	N10—C20—C22	125.8 (2)
C10—C11—N2	118.4 (2)	N10—C20—C21	115.8 (2)
C12—C11—N2	119.7 (2)	C22—C20—C21	118.4 (2)
C11—C12—C13	118.5 (2)	C20—C21—H21A	109.5
C11—C12—H12	120.8	C20—C21—H21B	109.5
C13—C12—H12	120.8	H21A—C21—H21B	109.5
C12—C13—C14	120.3 (3)	C20—C21—H21C	109.5
C12—C13—H13	119.8	H21A—C21—H21C	109.5
C14—C13—H13	119.8	H21B—C21—H21C	109.5
C13—C14—C9	120.6 (2)	C20—C22—H22A	109.5
C13—C14—H14	119.7	C20—C22—H22B	109.5
C9—C14—H14	119.7	H22A—C22—H22B	109.5
C15—N3—H3A	120.0	C20—C22—H22C	109.5
C15—N3—H3B	120.0	H22A—C22—H22C	109.5
H3A—N3—H3B	120.0	H22B—C22—H22C	109.5
C15—N4—H4A	120.0	H1W—O1W—H2W	112.0
C15—N4—H4B	120.0		
			
O2—C1—C2—C3	−178.0 (2)	C8—C9—C10—C11	179.0 (2)
O1—C1—C2—C3	0.3 (3)	C9—C10—C11—C12	0.2 (4)
O2—C1—C2—C7	0.6 (3)	C9—C10—C11—N2	179.9 (2)
O1—C1—C2—C7	178.9 (2)	O8—N2—C11—C10	−178.3 (2)
C7—C2—C3—C4	0.1 (3)	O7—N2—C11—C10	−0.1 (4)
C1—C2—C3—C4	178.8 (2)	O8—N2—C11—C12	1.4 (4)
C2—C3—C4—C5	1.0 (4)	O7—N2—C11—C12	179.6 (3)
C2—C3—C4—N1	−178.4 (2)	C10—C11—C12—C13	0.7 (4)
O3—N1—C4—C3	11.1 (4)	N2—C11—C12—C13	−178.9 (2)
O4—N1—C4—C3	−168.9 (3)	C11—C12—C13—C14	−0.5 (4)
O3—N1—C4—C5	−168.3 (3)	C12—C13—C14—C9	−0.6 (4)
O4—N1—C4—C5	11.7 (4)	C10—C9—C14—C13	1.5 (4)
C3—C4—C5—C6	−1.7 (4)	C8—C9—C14—C13	−178.8 (2)
N1—C4—C5—C6	177.7 (2)	C15—N5—N6—C16	−177.8 (2)
C4—C5—C6—C7	1.2 (4)	N6—N5—C15—N3	175.4 (2)
C5—C6—C7—C2	−0.2 (4)	N6—N5—C15—N4	−4.8 (3)
C3—C2—C7—C6	−0.5 (4)	N5—N6—C16—C18	−179.9 (2)
C1—C2—C7—C6	−179.1 (2)	N5—N6—C16—C17	−1.5 (4)
O6—C8—C9—C10	6.1 (3)	C19—N9—N10—C20	−179.9 (2)
O5—C8—C9—C10	−173.7 (2)	N10—N9—C19—N7	−6.3 (3)
O6—C8—C9—C14	−173.6 (2)	N10—N9—C19—N8	174.5 (2)
O5—C8—C9—C14	6.7 (4)	N9—N10—C20—C22	−1.7 (4)
C14—C9—C10—C11	−1.3 (4)	N9—N10—C20—C21	178.3 (2)

### Hydrogen-bond geometry (Å, °)

**Table:**

*D*—H···*A*	*D*—H	H···*A*	*D*···*A*	*D*—H···*A*
O1w—H1w···O1	0.84	2.06	2.878 (2)	164
O1w—H2w···O5	0.84	1.93	2.755 (2)	168
N3—H3A···O2	0.88	1.88	2.755 (3)	170
N3—H3B···O6^i^	0.88	2.01	2.812 (3)	150
N4—H4A···O1	0.88	2.06	2.943 (3)	177
N4—H4B···O5	0.88	2.32	3.049 (3)	139
N5—H5A···O6^i^	0.88	2.13	2.880 (3)	142
N7—H7A···O2^i^	0.88	2.04	2.779 (3)	141
N7—H7B···O1	0.88	2.07	2.831 (3)	144
N8—H8A···O1w^ii^	0.88	2.03	2.881 (3)	161
N8—H8B···O1w^iii^	0.88	2.13	2.975 (3)	162
N9—H9···O5^iii^	0.88	2.19	2.904 (3)	138
C5—H5···O7^iv^	0.95	2.58	3.322 (3)	135
C13—H13···O3^iii^	0.95	2.57	3.233 (4)	127
C21—H21B···O8^v^	0.98	2.34	3.290 (3)	164
C22—H22B···O6^vi^	0.98	2.57	3.538 (3)	168

Symmetry codes: (i) *x*, −*y*+3/2, *z*+1/2; (ii) *x*, *y*, *z*+1; (iii) −*x*+1, −*y*+2, −*z*+1; (iv) *x*+1, −*y*+3/2, *z*+1/2; (v) *x*+1, *y*, *z*; (vi) −*x*+1, −*y*+2, −*z*.
